# Neuroprotection in aortic arch surgery: a meta-analysis of hypothermia and selective cerebral perfusion on perioperative stroke and cognitive outcomes

**DOI:** 10.1097/MS9.0000000000003899

**Published:** 2025-09-24

**Authors:** Tirath Patel, Muhammad Farhan, Maral Daneshpazhouh, Ariana Seyfi, Alaa Musa Al Hawari, Odai Al Nahar, Abdulrhman Alkassar, Mazin Alazzavi, Yousif Al Ogaidi, Muhammad Hashir Nazir

**Affiliations:** a Trinity Medical Sciences University School of Medicine Saint Vincent and Grenadines, Ratho Mill, Kingstown; b College of Medicine, Ajman University, Ajman, United Arab Emirates; c College of Medicine, Gulf Medical University, Ajman, United Arab Emirates; d Department of Surgery, King Edward Medical University, Lahore, Pakistan

**Keywords:** aortic arch surgery, cognitive outcomes, hypothermia, meta-analysis, neuroprotection, selective cerebral perfusion, stroke

## Abstract

**Background:**

Hypothermic circulatory arrest is an essential aspect of aortic arch surgery and is classically used in severe hypothermia (≤20 °C) to reduce cerebral metabolic demand. However, severe hypothermia is associated with systemic problems. Recently, moderate hypothermia (20–28 °C) combined with selective cerebral perfusion (SCP) has been proposed as an option, which may reduce the risk of adverse neurological consequences. This study aims to see whether this newer approach confers any neuroprotective benefits.

**Methods:**

A comprehensive literature search was conducted on PubMed, Embase, Cochrane Library, Web of Science, and Scopus for English-language studies published in the last 20 years. Eligible studies comparing deep hypothermia with moderate hypothermia with SCP in adult patients after aortic arch surgery, with a focus on the incidence of perioperative stroke and neurocognitive outcomes. Data collection and quality assessment were performed using the Cochrane Risk of Bias Tool for Randomized Clinical Trials and the Newcastle-Ottawa Scale for observational studies. A meta-analysis of randomized clinical trials was performed using RevMan software.

**Results:**

Six studies (four randomized controlled trials and two observational studies) met the inclusion criteria. Pooled analysis indicated that deep hypothermia was associated with a significantly increased risk of perioperative stroke compared with moderate hypothermia (relative risk, 1.74; 95% confidence interval, 1.30–2.35), but heteogeneity (*I*^2^ = 0%). The funnel plot revealed a sand-synthesized model with minimal publication bias.

**Conclusion:**

Moderate hypothermia with SCP provides better neuroprotection than deep hypothermia in aortic arch surgery, which significantly reduces the incidence of perioperative stroke. Further in-depth studies are needed to validate these findings.

## Introduction

Hypothermic circulatory arrest (HCA) is crucial in major cardiovascular surgeries such as aortic arch surgery, protecting the brain and visceral organs from ischemic injury. The importance of hypothermia in this context lies in its ability to suppress cerebral metabolism and increase ischemic tolerance[1], as the brain is particularly susceptible to ischemia. The introduction of deep hypothermic cardiac arrest (DHCA) in the mid-1970s marked a significant advancement[2], enabling aortic arch surgery by reducing hypoxic tissues’ oxygen and metabolic requirements. Historically, this technique utilizes profound (≤14 °C) or deep (14.1–20.0 °C) hypothermia to maximize cerebral protection[3].

Despite the benefits of Hypothermia, it has drawbacks, as evidenced by clinical and laboratory research in recent years[4]. Profound hypothermia is associated with increased cardiopulmonary bypass time, coagulopathy, multi-organ dysfunction, systemic inflammatory response, endothelial dysfunction, and neuronal apoptosis^[5,6]^. These adverse effects contribute to worse neurological and renal outcomes, such as transient ischemic attacks, strokes, and kidney injuries, which significantly increase hospitalization costs and early mortality. Considering these concerns, the need for adjunctive techniques became apparent. Selective anterograde cerebral perfusion maintains physiological cerebral perfusion during circulatory arrest, providing neurological protection[7]. Additionally, there has been a trend towards using warmer core body temperatures during surgery, with positive outcomes observed[8]. Comparative analyses have indicated similar outcomes between deep (<20.0 °C)and moderate (20.1–28.0 °C) hypothermia, but significant variability in practice, and the optimal temperature for HCA is still debated, in part due to the lack of high-powered, multicenter, randomized controlled trials (RCTs).

This systematic review aimed to comprehensively evaluate the neurological effects of deep versus moderate hypothermia strategies during aortic arch surgery. This review seeks to reduce practice variability and establish evidence-based guidelines for perfusion strategies in aortic surgery by comparing the clinical outcomes between patients undergoing aortic arch surgery with mild versus moderate hypothermia. This review hypothesized that low–moderate hypothermia is non-inferior to deep hypothermia in terms of postoperative neurocognitive function, aiming to improve surgical outcomes and reduce the associated risks of HCA.

## Methods

The work has been reported in accordance with the Preferred Reporting Items for Systematic Reviews and Meta-Analyses (PRISMA)[9] and AMSTAR[10] guidelines. Before starting the review process, the methodology was defined and registered prospectively with PROSPERO.

### Data collection and search strategy

This systematic review and meta-analysis conducted a comprehensive literature search across multiple databases, including PubMed, Embase, the Cochrane Library, Web of Science, and Scopus. The search strategy was developed using terms related to aortic arch surgery and neuroprotection techniques, such as selective cerebral perfusion (SCP) and HCA. Boolean operators (“AND,” “OR”) and wildcard operators (“*/#”) were used to refine the search. The primary search terms included “cardiovascular surgery,” “coronary artery bypass graft,” “CABG,” “aortic arch surgery,” “aortic aneurysm,” “aortic dissection,” “selective cerebral perfusion,” “SCP,” “hypothermic circulatory arrest,” “HCA,” “stroke prevention,” “perioperative stroke,” “neuroprotection,” and “cognitive outcomes.” These search terms were used in all the databases and modified in each database accordingly via automated means.

### Selection criteria

Eligible studies included adult patients undergoing any major cardiovascular surgery with a high risk of stroke, regardless of sex or geographic location. We included studies focusing on HCA as a primary neuroprotective strategy, comparing DHCA (defined as intraoperative temperatures less than 20 °C) with moderate hypothermia (defined as intraoperative temperatures between 20 and 28 °C). The primary outcome of interest was the incidence of perioperative stroke (both ischemic and hemorrhagic). Only RCTs, cohort studies, and comparative studies published in peer-reviewed journals were considered. All studies had to have been published in English within the last 20 years. The 20-year publication window was chosen to ensure the inclusion of studies reflecting contemporary surgical techniques and neuroprotective strategies, as practices in aortic arch surgery and perfusion have evolved significantly over the past two decades.

Exclusion criteria included studies involving patients undergoing non-major cardiovascular surgeries or unrelated procedures, studies not using HCA as a primary intervention, and studies lacking a comparison group or those comparing methods irrelevant to neuroprotection in major cardiovascular surgery. We excluded studies that did not report stroke incidence, neurocognitive function, or post-surgical recovery, as well as case reports, editorials, reviews, and studies with fewer than 10 participants. Non-peer-reviewed publications and studies not published in English were also excluded.

**HIGHLIGHTS**

- The meta-analysis looked at the results of four randomized controlled trials (RCTs) and two observational studies to see how deep hypothermia (<20 °C) and moderate hypothermia (20–28 °C) with selective cerebral perfusion (SCP) affected patients who had aortic arch surgery.
- When compared to deep hypothermia, moderate hypothermia with SCP significantly decreased the risk of perioperative stroke (relative risk of 1.74, with a 95% confidence interval indicating statistical significance).
- There wasn’t much difference between the studies looked at (*I*² = 0%), suggesting that the results were the same across different research designs and settings.
- The quality assessments showed a low risk of bias in the RCTs, but the quality of the observational studies was variable. This indicated that the results of the meta-analysis could be trusted.
- The results indicate that moderate hypothermia and SCP can help protect nerve cells during surgery on the aortic arch. However, more large-scale studies should confirm and improve these results.

### Study endpoints and definitions

The primary outcome was the incidence of perioperative stroke (both ischemic and hemorrhagic). Outcome measures were assessed using standardized neurocognitive assessments and the National Cancer Institute Common Terminology Criteria for Adverse Events.

### Data extraction

All the records identified through the database search were uploaded to Rayyan.ai. The duplicates were detected and removed using the Rayyan tool. The remaining records were screened by their titles and abstracts to exclude irrelevant studies. The full texts of potentially eligible studies were assessed for eligibility. Two reviewers independently conducted screening and data extraction. Disagreements were resolved by discussion and consultation with the corresponding authors. Data were extracted into an Excel spreadsheet, and the following variables were collected: title of the article, year of publication, study design, characteristics of study arms, number of participants in each arm, age and sex of participants, information on prior chemotherapies, specifics of neuroprotective techniques, dosage and duration of neuroprotection, and outcome measures (efficacy and safety).

### Quality assessment

Quality assessments were conducted using standardized tools. For RCTs, the Cochrane Collaboration Risk of Bias (RoB) tool was used, which uses five different domains (randomization process, deviations from intended interventions, missing outcome data, measurement of the outcome, and selection of the reported result) to calculate the overall RoB in the article. For Cohort studies, the Newcastle–Ottawa Scale was used to assess quality. Each author then analyzed all selected articles, and the data were extracted and tabulated in the form of a spreadsheet using Google Docs. All data entries were cross-reviewed for errors.

### Statistical analyses

To measure the primary outcome, we used the weighted mean difference in the incidence of perioperative stroke between the Control and Placebo groups as an effect measure. The confidence interval was set at 95%. A Random Effects Meta-analysis was conducted using the “RevMan” software to obtain a forest plot. The Grading of Recommendations, Assessment, Development, and Evaluation (GRADE)[11] was used to assess the certainty of evidence. The tool grades the quality of evidence into one of four grades: high, moderate, low, and very low, depending on how close the actual effect might be to the estimated effect. A close association indicates a high quality of evidence and vice versa. Quality was judged for RoB, inconsistency of results, indirectness, imprecision, and publication bias. The chi-square test was used to assess the heterogeneity of the studies, and the values were interpreted according to the Cochrane Handbook of Systematic Reviews[12]. Inconsistency was quantified using the *I*^2^ index. Values greater than 50 were mainly considered inconsistent; however, the significance of said heterogeneity would depend on the number of studies included, as this was a random effects meta-analysis. For a smaller number of studies, performing subgroup analysis to explore any heterogeneity would not be worthwhile. Finally, a funnel plot was constructed to detect publication bias visually.

## Results

### Studies included

**Figure 1.**
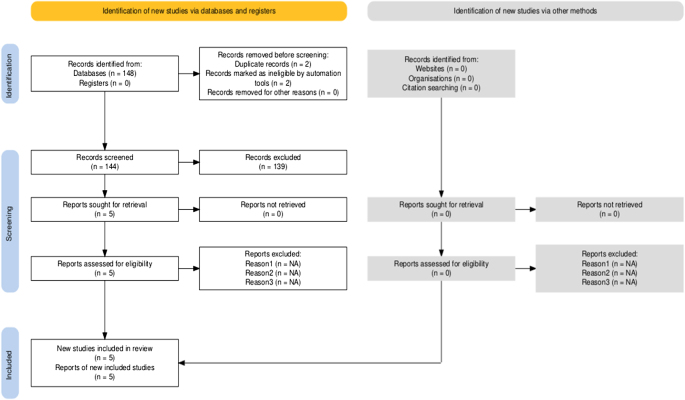
PRISMA flow diagram of the selection process. PRISMA, Preferred Reporting Items for Systematic Reviews and Meta-Analyses.

Two reviewers meticulously examined 144 articles from various databases after removing duplicates. Two investigators independently screened the titles of these publications. The full text was obtained when the titles met all the inclusion criteria and aligned with the research question. From the initial pool, 05 articles met the preliminary inclusion and exclusion criteria. All five articles were deemed the most relevant, and abstracts were available. Ultimately, we included the five studies with full texts in the results. The data extraction process is illustrated in the PRISMA flow chart 18 (Fig. 1).

### Study characteristics and reported outcomes

After thorough research, we included six studies in our systematic review. Four of these were RCTs, whereas two were observational studies. The characteristics of these studies are summarized in Table 1.

**Table 1:** Study characteristics and included outcomes

Study	Year	N1	N2	E1	E2	Type of study	Type of aortic arch or concomitant cardiovascular surgeries if performed	Additional cerebroprotective strategies
Algarni *et al*[13]	2019	75	53	16	7	Cohort/observational	Type A aortic dissection repair	Anterograde or retrograde cerebral perfusion
Hughes *et al*[14]	2023	97	4	179	6	RCT	Elective aortic arch surgery (hemi- or total arch) via median sternotomy	Anterior cerebral perfusion
Leshnower *et al*[15]	2019	11	1	9	1	RCT	Elective ascending aortic and hemiarch replacement	Retrograde or anterograde cerebral perfusion
Sanioglu *et al*[16]	2009	18	1	37	1	Cohort/observational	Supracoronary ascending aorta replacement, Bentall procedure, thoracic aorta replacement, total arch replacement and hemi-arch replacement	Antegrade cerebral perfusion
Sun *et al*[17]	2017	40	15	42	7	RCT	Stanford A aortic dissection surgery	
Kamenskaya *et al*[18]	2015	31	2	30	1	RCT	Pulmonary thromboendarterectomy	Antegrade cerebral perfusion and craniocerebral hypothermia

RCT, randomized controlled trial.

N1 = number of participants undergoing surgery with deep hypothermia, N2 = number of participants undergoing surgery with moderate hypothermia, E1 = number of events (perioperative stroke) in deep hypothermia group, E2 = number of events (perioperative stroke) in the moderate hypothermia group.

### Risk of bias assessment

The RoB tool, version 2.0, was used to assess the RoB in the included RCTs. The RoB (Table 2) was calculated across five different domains, which were then used to calculate the overall risk for each study using this tool. All the included trials had a low RoB (100%). Each study was independently checked for bias by two authors, and no discrepancies were found after mutual discussion. The Newcastle Ottawa Scale was used for cohort studies, which revealed that the study by Algarni *et al* was of high quality and scored well across all domains. In contrast, Sanioglu *et al* scored low, particularly regarding the selection of the non-exposed cohort and the comparability of cohorts. A table for the visual representation of the Newcastle Ottawa Scale is provided below (Table 3).

**Table 2:** Risk of bias for randomized controlled trials (RCTs)

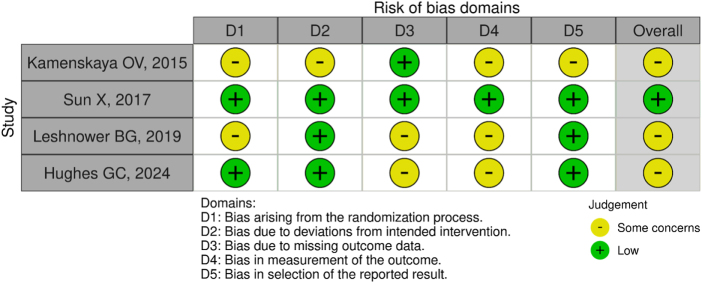

**Table 3:** **V**isual Representation of the Newcastle Ottawa Scale

Study ID	Representativeness of the exposed cohort	Selection of the non-exposed cohort	Ascertainment of exposure	Demonstration that outcome was not present at start	Comparability of cohorts based on study design or analysis	Assessment of outcome	Was follow-up long enough for outcomes to occur?	Adequacy of follow-up	Total stars
Algarni *et al*^[13]^	1	1	1	1	2	1	1	1	8/9
*et al*^[16]^	1	0	1	1	0	1	1	1	6/9

### Effect analysis

**Figure 2.**
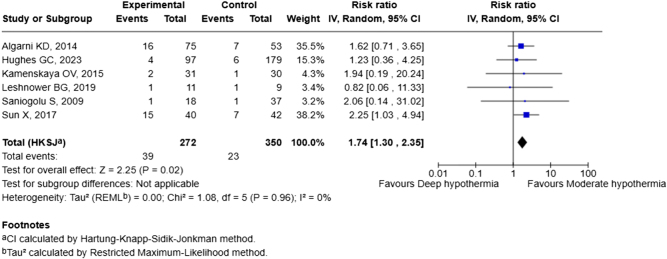
Forest plot comparing the effect of deep hypothermic circulatory arrest and moderate hypothermia in preventing the incidence of stroke. CI, confidence interval.

We constructed a forest plot (Fig. 2) to demonstrate the effect of deep hypothermic (<20 °C) circulatory arrest in preventing the incidence of stroke as compared to moderate hypothermia (20–28 °C). We found that DHCA was associated with a greater risk of developing perioperative stroke than moderate hypothermia [risk ratio (RR), 1.74; 95% CI (1.30, 2.35), *P* = 0.02]. No significant heterogeneity was observed, with an *I*^2^ value of 0%.

### Certainty assessment

Using the GRADE tool, we determined the certainty of the evidence to judge the incidence of perioperative stroke in both groups. We concluded this after judging the evidence for the RoB, imprecision, inconsistency, indirectness, and publication bias.

### Publication bias

**Figure 3.**
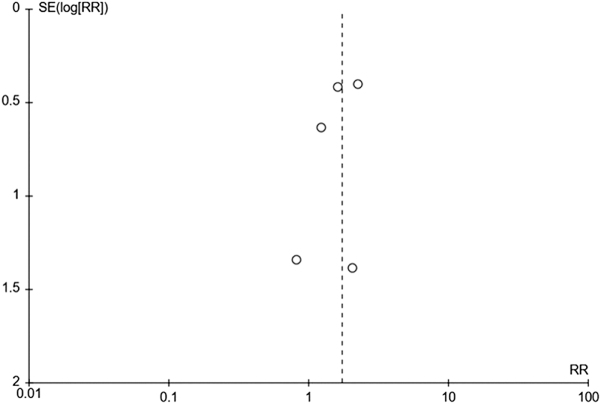
Funnel plot for publication bias. RR, risk ratio; SE, standard error.

A funnel plot (Fig. 3) was constructed, which showed high precision and a low risk of publication bias.

### Subgroup analysis

**Figure 4.**
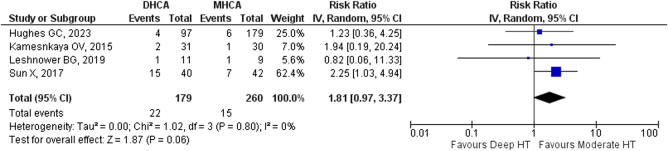
Funnel plot for subgroup analysis that includes only RCTs. HT, hypothermia; RCT, randomized controlled trial; DHCA, deep hypothermic cardiac arrest; MHCA, moderate deep hypothermic cardiac arrest.

Subgroup analysis (Fig. 4) was also performed, including only RCTs, in order to improve the strength of evidence and reduce bias. The results yielded no statistically significant benefit for moderate hypothermia (RR: 1.81, 95% CI: 0.97, 3.37, *P* = 0.08).

## Discussion

During major cardiovascular surgeries such as aortic arch surgery, hypothermia is employed to protect the brain and visceral organs from ischemic injury during periods of circulatory arrest. Lowering the body temperature reduces metabolic demands, thereby extending the safe duration of circulatory arrest. Traditionally, profound (≤14 °C) and deep (14.1–20.0 °C) hypothermia have been used to maximize cerebral metabolic suppression and ischemic tolerance[3]. However, concerns regarding the adverse effects of deep hypothermia, such as coagulopathy, systemic inflammatory response, and neuronal injury^[19,20]^, have led to the development of mild hypothermia strategies.

Primarily, the three major hypothermia strategies used in aortic arch surgery are deep hypothermia (≤20.0 °C), low-moderate hypothermia (20.1–24.0 °C), and high-moderate hypothermia (24.1–28.0 °C). Recent advancements have focused on moderate hypothermia combined with selective antegrade cerebral perfusion (ACP) to mitigate the risks associated with deep hypothermia while maintaining adequate cerebral protection in a near-physiological manner[21]. These newer strategies aim to balance the benefits of hypothermia with reduced systemic complications, leading to the possibility of a more extended period of circulatory arrest during these surgeries[22].

Recent trials, including the GOT ICE trial[14], have compared deep and moderate hypothermia during aortic arch surgery. The GOT ICE trial demonstrated that low-moderate hypothermia is non-inferior to deep hypothermia regarding global cognitive outcomes 4 weeks postoperatively. However, structured verbal memory was better preserved in the deep hypothermia group than in the high-moderate hypothermia group. The overall findings of the trial suggest that moderate hypothermia strategies can provide similar or even superior outcomes compared to traditional deep hypothermia.

Our meta-analysis found that moderate hypothermia significantly reduced the risk of postoperative stroke compared to deep hypothermia and was associated with better permanent neurologic outcomes. These results support the superiority of moderate hypothermia strategies, even in complex surgical cases that require prolonged neuroprotection. Implementing moderate hypothermia with ACP can enhance patient outcomes by reducing the risk of stroke and renal dysfunction while maintaining adequate cerebral protection. This approach allows for extended periods of safe circulatory arrest, making it suitable for complex aortic arch surgeries. However, it must be noted that a subgroup analysis of RCTs yielded no statistically significant benefit, possibly due to the small number of studies with a small sample size.

Our meta-analysis had several limitations. The heterogeneous nature of arch pathologies and variations in operative strategies across the studies may have influenced our results. Additionally, the inconsistent reporting of systemic outcomes, such as renal and hepatic dysfunction, limits the evaluation of the benefits of moderate hypothermia, which could not be assessed in this review. The lack of large-scale RCTs further restricts the generalizability of our findings. Future research should focus on standardized reporting and large-scale trials.

## Conclusion

In conclusion, our meta-analysis demonstrated that moderate hypothermia with ACP is a viable alternative to deep hypothermia in aortic arch surgery, offering comparable or superior outcomes. Moderate hypothermia strategies can improve patient outcomes and reduce systemic complications, making it a valuable approach for modern aortic arch surgery. Further in-depth studies are needed to validate these findings and improve neuroprotective techniques.

## Data Availability

The data used in this systematic review and meta-analysis were obtained from publicly available sources. The methodology section provides detailed search strategies, including keywords and inclusion criteria, to ensure transparency.

## Research registration unique identifying number (UIN)

1. Name of the registry: PROSPERO. 2. Registration number: CRD42025643542.
